# Association between regional critical care capacity and the incidence of invasive mechanical ventilation for coronavirus disease 2019: a population-based cohort study

**DOI:** 10.1186/s40560-024-00718-2

**Published:** 2024-01-30

**Authors:** Hiroyuki Ohbe, Satoru Hashimoto, Takayuki Ogura, Mitsuaki Nishikimi, Daisuke Kudo, Nobuaki Shime, Shigeki Kushimoto

**Affiliations:** 1 Department of Emergency and Critical Care Medicine, Tohoku University Hospital, 1-1 Seiryo-Machi, Aoba-Ku, Sendai, 980-8574 Japan; 2 Department of Clinical Epidemiology and Health Economics, School of Public Health, The University of Tokyo, 7-3-1 Hongo, Bunkyo-Ku, Tokyo, 113-0033 Japan; 3 Non-Profit Organization ICU Collaboration Network (ICON), Tokyo, Japan; 4 Tochigi Prefectural Emergency and Critical Care Centre, Imperil Gift Foundation SAISEIKAI, Utsunomiya Hospital, 911-1 Takebayashi-Machi, Utsunomiya, 321-0974 Japan; 5 Department of Emergency and Critical Care Medicine, Graduate School of Biomedical & Health Sciences, Hiroshima University, 1-2-3 Kasumi, Minami-Ku, Hiroshima, 734-8551 Japan; 6 Division of Emergency and Critical Care Medicine, Tohoku University Graduate School of Medicine, 2-1 Seiryo-Machi, Aoba-Ku, Sendai, Miyagi 980-8575 Japan

**Keywords:** COVID-19, Invasive mechanical ventilation, Critical care capacity, Population study, Intensive care unit

## Abstract

**Background:**

Coronavirus disease 2019 (COVID-19) has exposed critical care supply shortages worldwide. This study aimed to investigate the association between regional critical care capacity and the incidence of invasive mechanical ventilation following novel COVID-19 during the pandemic in Japan, a country with a limited intensive care unit (ICU) bed capacity of a median of 5.1 ICU beds per 100,000 individuals.

**Methods:**

This population-based cohort study used data from the CRoss Icu Searchable Information System database and publicly available databases provided by the Japanese government and Japanese Society of Intensive Care Medicine. We identified patients recently diagnosed with COVID-19, those who received invasive mechanical ventilation, and those who received extracorporeal membrane oxygenation (ECMO) between February 2020 and March 2023. We analyzed the association between regional critical care capacity (ICU beds, high-dependency care unit (HDU) beds, resource-rich ICU beds, and intensivists) and the incidence of invasive mechanical ventilation, ECMO, and risk-adjusted mortality across 47 Japanese prefectures.

**Results:**

Among the approximately 127 million individuals residing in Japan, 33,189,809 were recently diagnosed with COVID-19, with 12,203 and 1,426 COVID-19 patients on invasive mechanical ventilation and ECMO, respectively, during the study period. Prefecture-level linear regression analysis revealed that the addition of ICU beds, resource-rich ICU beds, and intensivists per 100,000 individuals increased the incidence of IMV by 5.37 (95% confidence interval, 1.99–8.76), 7.27 (1.61–12.9), and 13.12 (3.48–22.76), respectively. However, the number of HDU beds per 100,000 individuals was not statistically significantly associated with the incidence of invasive mechanical ventilation. None of the four indicators of regional critical care capacity was statistically significantly associated with the incidence of ECMO and risk-adjusted mortality.

**Conclusions:**

The results of prefecture-level analyses demonstrate that increased numbers of ICU beds, resource-rich ICU beds, and intensivists are associated with the incidence of invasive mechanical ventilation among patients recently diagnosed with COVID-19 during the pandemic. These findings have important implications for healthcare policymakers, aiding in efficiently allocating critical care resources during crises, particularly in regions with limited ICU bed capacities.

*Registry and the registration no. of the study/trial* The approval date of the registry was August 20, 2020, and the registration no. of the study was lUMIN000041450.

**Supplementary Information:**

The online version contains supplementary material available at 10.1186/s40560-024-00718-2.

## Background

Invasive mechanical ventilation (IMV) plays a crucial role in sustaining the lives of critically ill patients. Guidelines for admitting patients to the intensive care unit (ICU) recommend that those requiring IMV should receive care in an ICU rather than a high-dependency care unit (HDU) or general ward [1, 2]. This recommendation is supported by studies demonstrating that ICU care for mechanically ventilated patients with and without coronavirus disease 2019 (COVID-19) is associated with lower in-hospital mortality than care in the HDU or general ward [3, 4].

The novel COVID-19 has exposed critical care supply shortages worldwide, including those in critical care beds, hospital staff, and mechanical ventilators [5, 6]. In several regions, critically ill COVID-19 patients overflowed from ICUs into HDUs, post-anesthesia care units, emergency departments, operating rooms, general wards, and even temporary or tent facilities, where they may not have received appropriate critical care [7]. A previous study in the United States revealed that up to 25% of COVID-19 deaths could be attributed to surges in the COVID-19 caseload and loss of access to critical care [8].

Significant differences exist in regional critical care capacity between and within countries worldwide. Countries with high ICU bed capacity include the United States (34.7 ICU beds per 100,000 individuals), Germany (29.2 ICU beds), and Taiwan (28.5 ICU beds) [9–11]. In contrast, a recent study estimated that at least 96 countries, particularly those identified as low- and middle-income countries, exhibit a density of less than 5.0 ICU beds per 100,000 individuals [12–16].

Critically ill COVID-19 patients in regions with insufficient critical care capacity may not receive appropriate critical care. A study analyzing hospital-level critical care capacity across the United States found that a low number of ICU beds was associated with increased COVID-19 mortality [17]. Other studies in the United States found that higher ICU bed occupancy in the hospital was associated with higher COVID-19 mortality [18, 19]. However, the effects of regional critical care capacity on COVID-19 patients who require IMV remain unclear.

Therefore, this study aimed to examine the association between regional critical care capacity and the incidence of IMV for COVID-19 patients in Japan, a country with a limited ICU bed capacity of a median of 5.1 ICU beds per 100,000 individuals. We assumed that a comparable number of COVID-19 patients would develop acute critical illness requiring IMV in different regions. Our findings will indicate the optimal critical care capacity for the population at the regional level within a country during the pandemic and contribute to planning the most efficient regional critical care system.

## Methods

### Study design and data collection

We aimed to evaluate the association between regional critical care capacity and the incidence of IMV for COVID-19 patients in Japan. This population-based cohort study included all Japanese citizens. The data used in this study were obtained from three sources: (i) CRoss Icu Searchable Information System (CRISIS) database; (ii) publicly available databases provided by the Japanese government; and (iii) published data from the Japanese Society of Intensive Care Medicine.

The CRISIS database was developed by Japan ECMOnet for COVID-19 to accurately track real-time information from ICUs across Japan during the COVID-19 pandemic from February 2020 [20–23]. Participation in the CRISIS database was voluntary for hospitals; however, most hospitals certified by the Japanese Society of Intensive Care Medicine and Japanese Association for Acute Medicine participated initially. Hence, there were 666 participating hospitals with 6186 ICU beds and 10,058 HDU beds in the database as of March 31, 2023. As of July 2020, participating hospitals comprised 86.7% of all the ICU beds (6,181/7,132 beds) and 71% of all the HDU beds (10,058/13,546 beds) in Japan, indicating that most hospitals with critical care beds in Japan participated in the CRISIS database. Participating hospitals were requested to register all COVID-19 patients who received IMV and extracorporeal membrane oxygenation (ECMO) in the CRISIS database. In the CRISIS database, COVID-19 patients were defined as those with a positive severe acute respiratory syndrome coronavirus-2 polymerase chain reaction test result and pulmonary involvement typical of COVID-19. The CRISIS database collected data on the number of patients who received IMV and ECMO, hospital characteristics, patient background (age, sex, and body mass index), start and end days of IMV and ECMO, and mortality.

Three publicly available databases from the Japanese government were used: trends in newly confirmed COVID-19 cases [24], Survey of Medical Institution 2020 [25], and Japanese Population Estimates 2020 [26]. The number of newly confirmed cases is provided by the Ministry of Health, Labour and Welfare, Japan, and is calculated based on the Health Center Real-time Information-sharing System on COVID-19 (HER-SYS) database [27]. HER-SYS allows information to be shared instantly among local public health centers, prefectures, municipalities, medical institutions, and other relevant parties. In the HER-SYS database, newly confirmed cases of COVID-19 were defined as those diagnosed with COVID-19 by physicians, regardless of the diagnostic method. The number of newly confirmed cases, including recurrent positive cases in each prefecture, is calculated by summing the cases published through press releases. The Survey of Medical Institutions 2020 is provided by the Ministry of Health, Labour and Welfare, Japan, and includes facility information and statistics on all hospitals in Japan as of July 1, 2020. The Survey of Medical Institutions included data on the types of wards (e.g., ICU, HDU, and resource-rich ICU) and the number of beds in each ward for 47 prefectures in Japan. The Japanese Population Estimates 2020 is provided by the Ministry of Internal Affairs and Communications. The annual estimates of “population by age (5-year groups) and sex for prefectures” as of October 1, 2020, were used in this study.

The number of intensivists certified by the Japanese Society of Intensive Care Medicine in each of the 47 prefectures as of April 1, 2021, was obtained using data published by the Japanese Society of Intensive Care Medicine [28].

### Study and patient populations

The patient population included COVID-19 patients diagnosed between February 1, 2020, and March 31, 2023, as recorded in the HER-SYS and CRISIS databases. Patients with missing data on age at admission or sex in the CRISIS database were excluded. All eligible patients in the CRISIS database were followed up until death, hospital transfer, or hospital discharge.

### Regional critical care capacity

The variable of interest in this study was the regional critical care capacity of 47 prefectures in Japan. Prefectures in Japan are administrative divisions similar to those in the United States, and medical plans stipulate the formulation of critical care capacity systems at the prefectural level. To assess regional critical care capacity, we used four indicators: (i) numbers of ICU beds, (ii) HDU beds, (iii) resource-rich ICU beds, and (iv) board-certified intensivists per 100,000 individuals. The numbers of ICU beds, HDU beds, and resource-rich ICU beds were obtained from the Survey of Medical Institutions 2020, and the number of intensivists was obtained from published data from the Japanese Society of Intensive Care Medicine. When calculating these indicators, the population of each of the 47 prefectures was standardized to the total population in 2020 based on age (5-year groups) and sex.

### Definition of critical care beds

In Japan, the ICU is defined as a separate unit providing critical care services with at least one physician onsite 24 h per day; full-time, around-the-clock nursing; equipment necessary to care for critically ill patients; and a nurse-to-patient ratio of 1:2 [1, 29]. The term “ICU” includes all ICU types, including medical, surgical, medical-surgical, emergency, neuro, cardiac, and pediatric ICUs. An HDU, also called an “intermediate care unit” or “step down unit”, is defined as an area where critical care services (mechanical ventilation and vasopressor administration) are provided, with patient care levels between those of the ICU and the general wards [30, 31]. In this study, the HDU differed from the ICU because it had a nurse-to-patient ratio of 1:4 or 1:5 and did not require intensive staffing [29]. Among the ICUs defined previously, a resource-rich ICU was defined as ICUs with two or more intensivists working as full-time employees, ≥ 20 m^2^ per ICU bed, and a medical engineer in the hospital 24 h per day [32]. The medical reimbursement for resource-rich ICUs is 1.5 times that for other ICUs. The details of the Japanese procedure codes used to define care beds are shown in Additional file 1: Table S1.

## Outcomes

The three study outcomes for each of the 47 prefectures were as follows: (i) incidence of IMV per 100,000 newly diagnosed COVID-19 patients; (ii) incidence of ECMO per 100,000 newly diagnosed COVID-19 patients; and (iii) risk-adjusted mortality of COVID-19 patients who received IMV. The proportion of CRISIS-participating hospitals varied across prefectures and ranged from 44.4 to 100% regarding ICU beds, as shown in Additional file 1: Table S2. Therefore, to account for this variation when calculating the incidence, the number of patients requiring IMV and ECMO was divided by the proportion of the total ICU beds in CRISIS-participating hospitals to the total number of ICU beds in the Survey of Medical Institution in each prefecture. Subsequently, the incidence was calculated by dividing the estimated number of patients requiring IMV and ECMO by the number of newly diagnosed COVID-19 cases. When calculating the risk-adjusted standardized mortality rate of COVID-19 patients on IMV, we initially estimated the individual risk of mortality for each patient on IMV using multivariate logistic regression adjusted for 5-year age category, sex, body mass index category, and outbreaks of COVID-19 on the day of initiation of IMV. Subsequently, we calculated the expected number of deaths in each prefecture by summing the probabilities for each patient within the prefecture. Finally, the prefecture-specific risk-adjusted mortality was calculated by multiplying the ratio of the observed to the expected number of deaths in each prefecture by the overall crude mortality rate. Based on a report from the Ministry of Health, Labour and Welfare, Japan, we categorized eight outbreaks of COVID-19, starting in February 2020, June 2020, November 2020, April 2021, July 2021, January 2022, July 2022, and November 2022 [33].

### Statistical analyses

The prefecture-level associations between the four indicators of regional critical care capacity and the three study outcomes were illustrated and analyzed using linear regression analyses. Following Hansen’s methods [34, 35], one or more potential switch points in the association between regional critical care capacity and study outcome were identified endogenously from the data rather than arbitrary, using the command “threshold” in STATA [36]. A correlation matrix was prepared for the four indicators of regional critical care capacity. All reported p-values were two-sided, and p-values < 0.05 were considered statistically significant. All statistical analyses were performed using the STATA/SE version 17.0 software (STATA, College Station, TX, USA).

### Sensitivity analyses

We conducted two sensitivity analyses. First, to account for the regional-level confounders, we performed linear regression analyses adjusted for the numbers of emergency physicians, pulmonologists, nurses, and clinical engineers per 100,000 individuals in the regions. The number of emergency physicians and pulmonologists was obtained from Statistics of Physicians, Dentists, and Pharmacists 2020 [37], while the numbers of nurses and clinical engineers were obtained from the Survey of Medical Institutions 2020. Second, we performed multi-level generalized linear models on patient-level mortality, with mortality as the dependent variable. The model included four indicators of regional critical care capacity, 5-year age category, sex, body mass index category, and outbreaks of COVID-19 on the day of initiation of IMV as independent variables, with prefecture as a random intercept and an identity link function. These multi-level analyses were specifically performed for mortality among the three study outcomes.

## Results

### Regional critical care capacity

The overall population in Japan was 126,146,000, and the median population of the 47 prefectures was 1,590,000, with an interquartile range of 1,068,000–2,798,000 (Table 1). The median number of ICU beds per 100,000 individuals in the 47 prefectures was 5.1 (interquartile range, 3.9–6.3), with the lowest in Niigata Prefecture at 1.5 and the highest in Okayama Prefecture at 11.8. The median number of HDU beds per 100,000 individuals was 10.2 (interquartile range, 8.6–12.2 [minimum, 4.9; maximum, 20.5]). The median number of resource-rich ICU beds per 100,000 individuals was 1.7 (interquartile range, 1.0–2.8 [minimum, 0.3; maximum, 5.8]). The median number of intensivists per 100,000 individuals was 1.7 (interquartile range, 1.0–2.0 [minimum, 0.5; maximum, 3.5]). The correlation matrices of the four indicators of regional critical care capacity are presented in Additional file 1: Table S3.

**Table 1:** ICU, HDU, resource-rich ICU beds, and intensivists per 100,000 individuals in 47 Japanese prefectures

Prefectures	Population, person	Number of ICU beds, beds	Number of HDU beds, beds	Number of resource-rich ICU beds, beds	Number of intensivists, person	Number of ICU beds, beds per 100,000 population	Number of HDU beds, beds per 100,000 population	Number of resource-rich ICU beds, beds per 100,000 population	Number of intensivists, person per 100,000 population
Overall	126146000	7132	13546	2445	2115	5.7	10.7	1.9	1.7
Hokkaido	5225000	221	647	100	83	4.2	12.4	1.9	1.6
Aomori	1238000	68	71	16	12	5.5	5.7	1.3	1.0
Iwate	1211000	32	116	8	10	2.6	9.6	0.7	0.8
Miyagi	2302000	126	185	66	41	5.5	8.0	2.9	1.8
Akita	960000	36	76	16	7	3.7	7.9	1.7	0.7
Yamagata	1068000	32	106	6	14	3.0	9.9	0.6	1.3
Fukushima	1833000	116	171	28	17	6.3	9.3	1.5	0.9
Ibaraki	2867000	140	206	16	25	4.9	7.2	0.6	0.9
Tochigi	1933000	95	192	58	36	4.9	9.9	3.0	1.9
Gumma	1939000	75	215	53	29	3.9	11.1	2.7	1.5
Saitama	7345000	327	771	75	82	4.5	10.5	1.0	1.1
Chiba	6284000	400	569	74	114	6.4	9.1	1.2	1.8
Tokyo	14048000	1103	1472	317	303	7.9	10.5	2.3	2.2
Kanagawa	9237000	480	1022	84	153	5.2	11.1	0.9	1.7
Niigata	2201000	32	174	8	11	1.5	7.9	0.4	0.5
Toyama	1035000	30	79	8	35	2.9	7.6	0.8	3.4
Ishikawa	1133000	52	158	28	15	4.6	13.9	2.5	1.3
Fukui	767000	37	113	20	10	4.8	14.7	2.6	1.3
Yamanashi	810000	12	40	12	17	1.5	4.9	1.5	2.1
Nagano	2048000	105	420	28	13	5.1	20.5	1.4	0.6
Gifu	1979000	74	191	42	19	3.7	9.7	2.1	1.0
Shizuoka	3633000	148	378	38	36	4.1	10.4	1.0	1.0
Aichi	7542000	403	768	208	137	5.3	10.2	2.8	1.8
Mie	1770000	43	164	6	17	2.4	9.3	0.3	1.0
Shiga	1414000	54	104	12	26	3.8	7.3	0.8	1.8
Kyoto	2578000	161	355	100	68	6.2	13.8	3.9	2.6
Osaka	8838000	647	1077	272	178	7.3	12.2	3.1	2.0
Hyogo	5465000	371	567	120	101	6.8	10.4	2.2	1.8
Nara	1324000	83	160	40	24	6.3	12.1	3.0	1.8
Wakayama	923000	61	105	10	9	6.6	11.4	1.1	1.0
Tottori	553000	26	82	6	5	4.7	14.8	1.1	0.9
Shimane	671000	41	55	28	19	6.1	8.2	4.2	2.8
Okayama	1888000	223	190	77	66	11.8	10.1	4.1	3.5
Hiroshima	2800000	101	241	42	48	3.6	8.6	1.5	1.7
Yamaguchi	1342000	84	119	28	31	6.2	8.8	2.1	2.3
Tokushima	720000	34	85	11	13	4.7	11.8	1.5	1.8
Kagawa	950000	66	144	28	20	6.9	15.1	2.9	2.1
Ehime	1335000	78	129	12	15	5.8	9.6	0.9	1.1
Kochi	692000	54	139	40	21	7.8	20.1	5.8	3.0
Fukuoka	5135000	340	748	92	80	6.6	14.6	1.8	1.6
Saga	811000	40	105	18	13	4.9	12.9	2.2	1.6
Nagasaki	1312000	68	142	20	15	5.2	10.8	1.5	1.1
Kumamoto	1738000	89	220	51	34	5.1	12.7	2.9	2.0
Oita	1124000	44	92	8	20	3.9	8.2	0.7	1.8
Miyazaki	1070000	50	57	16	15	4.7	5.3	1.5	1.4
Kagoshima	1588000	90	158	40	27	5.7	9.9	2.5	1.7
Okinawa	1467000	140	168	59	31	9.5	11.4	4.0	2.1

*ICU* intensive care unit; *HDU* high-dependency care unit; *IMV* invasive mechanical ventilation

## Outcomes

Between February 1, 2020, and March 31, 2023, 33,189,809 newly confirmed COVID-19 cases were identified in the HER-SYS database (Table 2). During the same period, 12,203 COVID-19 patients receiving IMV from 322 hospitals and 1,426 COVID-19 patients receiving ECMO from 193 hospitals were identified in the CRISIS database. The overall incidence of IMV per 100,000 COVID-19 patients was 36.8, and the median incidence was 21.7 (interquartile range, 12.6–42.7), with the lowest in Iwate Prefecture at 1.3 and the highest in Osaka Prefecture at 124.4. The overall incidence of ECMO per 100,000 COVID-19 patients was 4.3, and the median incidence was 3.2 (interquartile range, 1.1–5.8 [minimum, 0.0; maximum, 11.1]).

**Table 2:** IMV and ECMO incidence and risk-adjusted mortality in COVID-19 patients in 47 Japanese prefectures

Prefectures	Number of COVID-19 patients, n	Number of IMV patients, n	Number of ECMO patients, n	Mortality of COVID-19 with IMV, n	Incidence of IMV, per 100,000 COVID-19 patients	Incidence of ECMO, per 100,000 COVID-19 patients	Risk-adjusted mortality of IMV patients, %
Overall	33189809	12203	1426	2706	36.8	4.3	22.2
Hokkaido	1320084	394	50	81	53.2	6.8	21.3
Aomori	276472	32	17	5	14.6	7.7	18.5
Iwate	234363	3	1	1	1.3	0.4	33.6
Miyagi	537709	115	17	22	21.4	3.2	18.2
Akita	199402	25	4	14	12.5	2.0	54.9
Yamagata	221470	27	7	9	17.7	4.6	32.9
Fukushima	402079	47	11	13	14.4	3.4	29.0
Ibaraki	637659	126	28	26	23.4	5.2	21.4
Tochigi	414530	224	35	32	54.0	8.4	16.0
Gumma	437463	171	31	30	39.1	7.1	18.7
Saitama	1804519	671	105	169	39.4	6.2	26.3
Chiba	1444324	677	139	164	54.2	11.1	25.4
Tokyo	4317391	1840	224	469	49.6	6.0	26.0
Kanagawa	2173335	700	128	193	34.3	6.3	27.4
Niigata	455803	62	3	9	18.1	0.9	14.8
Toyama	234869	47	5	12	30.0	3.2	24.8
Ishikawa	278895	83	8	20	40.7	3.9	23.0
Fukui	200593	80	3	12	39.9	1.5	14.6
Yamanashi	191124	24	11	11	12.6	5.8	41.9
Nagano	459377	43	4	7	11.0	1.0	16.6
Gifu	537974	103	14	15	22.1	3.0	17.6
Shizuoka	857104	72	2	13	9.0	0.3	18.4
Aichi	2109638	421	67	111	21.7	3.4	26.9
Mie	460378	77	5	22	19.4	1.3	32.8
Shiga	373039	124	19	32	42.7	6.5	25.3
Kyoto	670867	500	65	86	74.5	9.7	16.8
Osaka	2783907	3008	192	560	124.4	7.9	17.5
Hyogo	1438949	666	35	196	59.6	3.1	28.6
Nara	339170	143	14	23	51.5	5.0	15.3
Wakayama	234436	28	3	7	11.9	1.3	21.9
Tottori	140333	20	0	1	14.3	0.0	5.4
Shimane	168610	12	1	4	7.9	0.7	40.1
Okayama	488069	265	8	41	68.0	2.1	15.9
Hiroshima	806496	136	18	34	18.7	2.5	28.5
Yamaguchi	313686	58	7	10	18.5	2.2	18.4
Tokushima	166702	98	3	12	58.8	1.8	12.5
Kagawa	252309	13	0	3	5.2	0.0	23.2
Ehime	315245	25	3	6	9.1	1.1	25.7
Kochi	167800	20	2	9	13.4	1.3	40.0
Fukuoka	1560629	320	86	80	25.2	6.8	28.8
Saga	259106	18	2	7	6.9	0.8	40.5
Nagasaki	331994	27	2	2	9.9	0.7	7.4
Kumamoto	531201	130	2	26	27.6	0.4	21.2
Oita	302416	30	3	10	10.9	1.1	30.4
Miyazaki	319954	65	2	11	20.3	0.6	16.6
Kagoshima	439967	75	10	22	38.4	5.1	26.6
Okinawa	578369	358	30	64	66.7	5.6	17.4

Mortality was adjusted for 5-year age category, sex, body mass index category, and outbreaks of COVID-19

COVID-19, coronavirus disease 2019

*IMV* invasive mechanical ventilation; *ECMO* extracorporeal membrane oxygenation

Among the 12,203 COVID-19 patients receiving IMV, the mean age was 63.1 years (standard deviation, 14.8 years), and 74.5% were male (Table 3). Overall, 17.1% of the patients were obese (body mass index ≥ 30.0 kg/m^2^). The proportion of patients who received ECMO was 11.7%. The overall mortality rate of COVID-19 patients receiving IMV was 22.2% (*n* = 2706/12,203). The median risk-adjusted mortality rate of COVID-19 patients requiring IMV was 22.2% (interquartile range, 17.3–28.6% [minimum, 5.4%; maximum, 54.9%]) (Table 2).

**Table 3:** Characteristics and outcomes of COVID-19 patients requiring invasive mechanical ventilation

Variables	Total
	*N* = 12,203
Age, years, mean (SD)	63.1 (14.8)
Age category, years, *n* (%)	
0–4	65 (0.5%)
5–9	30 (0.2%)
10–14	27 (0.2%)
15–19	34 (0.3%)
20–24	53 (0.4%)
25–29	115 (0.9%)
30–34	155 (1.3%)
35–39	251 (2.1%)
40–44	433 (3.5%)
45–49	803 (6.6%)
50–54	1152 (9.4%)
55–59	1408 (11.5%)
60–64	1422 (11.7%)
65–69	1600 (13.1%)
70–74	1904 (15.6%)
75–79	1453 (11.9%)
80–84	853 (7.0%)
85-	445 (3.6%)
Male, n (%)	9089 (74.5%)
Body mass index, kg/m^2^, n (%)	
< 18.5	583 (4.8%)
18.5–24.9	4730 (38.8%)
25.0–29.9	3558 (29.2%)
≥ 30.0	2084 (17.1%)
Missing	1248 (10.2%)
Outbreaks of COVID-19	
1st (from February 2020)	817 (6.7%)
2nd (from June 2020)	895 (7.3%)
3rd (from November 2020)	3077 (25.2%)
4th (from April 2021)	2554 (20.9%)
5th (from July 2021)	2602 (21.3%)
6th (from January 2022)	1087 (8.9%)
7th (from July 2022)	614 (5.0%)
8th (from November 2022)	557 (4.6%)
Extracorporeal membrane oxygenation, *n* (%)	1426 (11.7%)
Mortality, *n* (%)	2706 (22.2%)

*COVID-19* novel coronavirus disease; *SD* standard deviation

### Association between indicators of regional critical care capacity and study outcomes

**Fig. 1:**
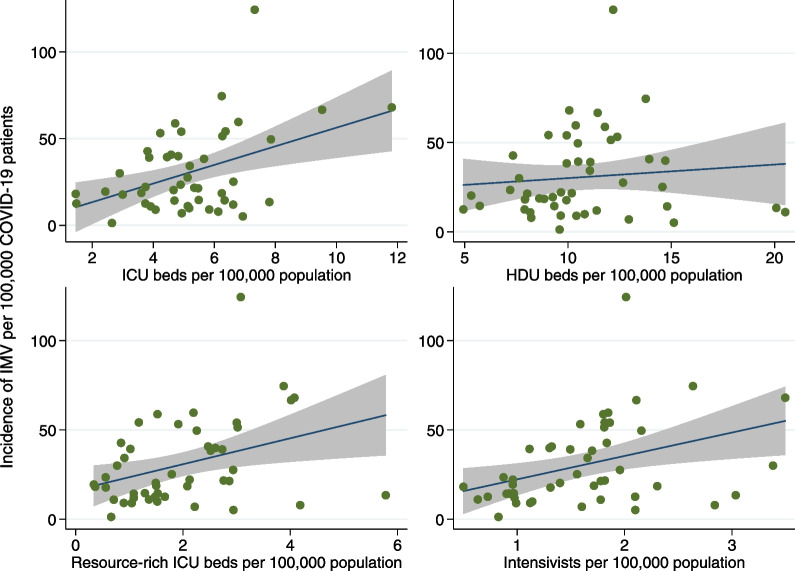
Association between the incidence of invasive mechanical ventilation and four indicators of critical care capacity. Association between the incidence of invasive mechanical ventilation per 100,000 COVID-19 patients and four indicators of critical care capacity, including numbers of ICU beds, HDU beds, resource-rich ICU beds, and intensivists per 100,000 individuals. *IMV* invasive mechanical ventilation; *COVID-19* coronavirus disease 2019; *ICU* intensive care unit; *HDU* high-dependency care unit

**Fig. 2:**
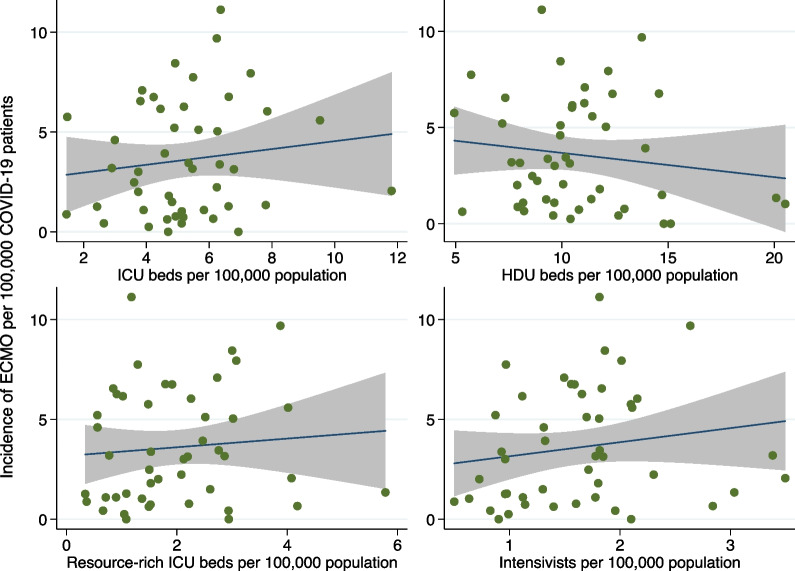
Association between the incidence of ECMO and four indicators of critical care capacity. Association between the incidence of ECMO per 100,000 COVID-19 patients and four indicators of critical care capacity, including numbers of ICU beds, HDU beds, resource-rich ICU beds, and intensivists per 100,000 individuals. *ECMO* extracorporeal membrane oxygenation; *COVID-19* coronavirus disease 2019; *ICU* intensive care unit; *HDU* high-dependency care unit

**Fig. 3:**
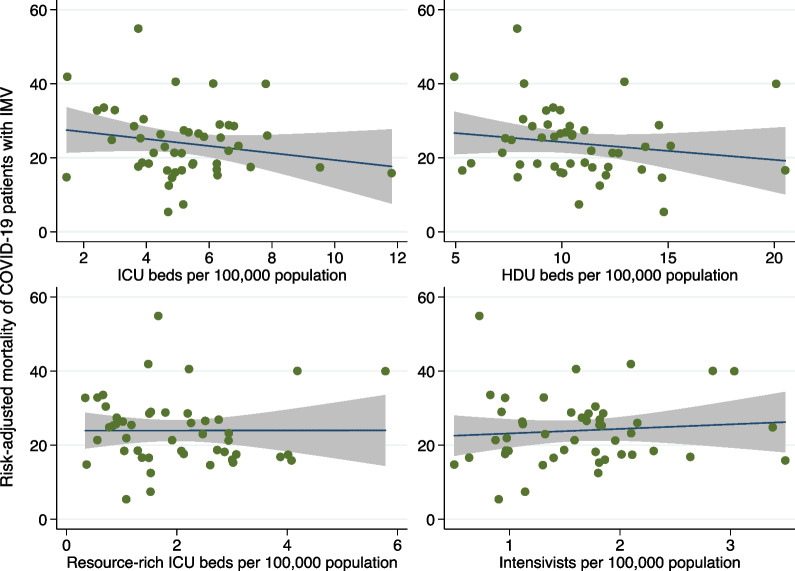
Association between the risk-adjusted mortality and four indicators of critical care capacity. Association between the risk-adjusted mortality of COVID-19 patients with invasive mechanical ventilation and four indicators of critical care capacity, including numbers of ICU beds, HDU beds, resource-rich ICU beds, and intensivists per 100,000 individuals. Mortality was adjusted for 5-year age category, sex, body mass index category, and outbreaks of COVID-19. *IMV* invasive mechanical ventilation; *COVID-19* coronavirus disease 2019; *ICU* intensive care unit; *HDU* high-dependency care unit

Linear regression analysis revealed that additional ICU beds, resource-rich ICU beds, and intensivists per 100,000 individuals increased the incidence of IMV by 5.37 (Table 4 and Fig. 1; 95% confidence interval [CI] 1.99–8.76; *p* = 0.003), 7.27 (95% CI 1.61–12.9; *p* = 0.013), and 13.12 (95% CI 3.48–22.76; *p* = 0.009), respectively. However, the number of HDU beds per 100,000 individuals was not statistically significantly associated with the incidence of IMV. No statistically significant association existed between the four indicators of regional critical care capacity and the incidence of ECMO (Table 4 and Fig. 2) and risk-adjusted mortality (Table 4 and Fig. 3). The switch point analyses detected only one switch point in all analyses (Additional file 1: Table S4). In the statistically significant associations with the incidence of IMV in the linear regression analyses, the switch points for those with ICU beds, resource-rich ICU beds, and intensivists were 6.93 beds, 3.01 beds, and 2.01 persons per 100,000 population, respectively.

**Table 4:** Association between the four indicators of regional critical care capacity and the three study outcomes

Number of regional critical care capacity per 100,000 population	Outcomes					
	Incidence of IMV per 100,000 COVID-19		Incidence of ECMO per 100,000 COVID-19		Risk-adjusted mortality (%)	
	Coef. (95% CI)	*P*-value	Coef. (95%CI)	*P*-value	Coef. (95%CI)	*P*-value
ICU beds	5.37 (1.99, 8.76)	0.003	0.20 (− 0.25, 0.65)	0.383	− 0.95 (− 2.41, 0.51)	0.196
HDU beds	0.76 (− 1.47, 2.99)	0.497	− 0.13 (− 0.39, 0.14)	0.350	− 0.48 (− 1.36, 0.40)	0.276
Resource-rich ICU beds	7.27 (1.61, 12.9)	0.013	0.22 (− 0.51, 0.95)	0.552	0.01 (− 2.39, 2.41)	0.994
Intensivists	13.12 (3.48, 22.76)	0.009	0.71 (− 0.54, 1.95)	0.258	1.23 (− 2.88, 5.34)	0.551

*ICU* intensive care unit; *HDU* high-dependency care unit; *IMV* invasive mechanical ventilation; *COVID-19* coronavirus disease 2019; *ECMO* extracorporeal membrane oxygenation; *CI* confidence interval

## Results of sensitivity analyses

The results of sensitivity analyses, which further adjusted for regional-level confounders, were similar to those of the main analyses (Additional file 1: Table S5). The sensitivity analyses conducted with multi-level generalized linear models produced results consistent with the main analyses and were not statistically significant (Additional file 1: Table S6).

## Discussion

A nationwide population-based cohort study was performed in Japan, where the number of ICU beds and intensivists per 100,000 individuals was considerably lower than that in countries with high critical care capacity. The prefecture-level analyses demonstrated that higher numbers of ICU beds, resource-rich ICU beds, and intensivists per 100,000 individuals were associated with a higher incidence of IMV for COVID-19 patients, but a high number of HDU beds per 100,000 individuals did not show a significant association. No significant associations were found between the regional critical care capacity indicators and the incidence of ECMO or risk-adjusted mortality.

In Japan, the median number of ICU beds per 100,000 individuals is 5.1 (minimum, 1.5; maximum, 11.8), with a significant variation of up to 7.9 times across the 47 regions within the country. Even in regions with high ICU bed capacity in Japan, the number of ICU beds is relatively low compared to that of the ICU beds in countries with high critical care capacity [9–11]. This is also below the average of 12.1 in the 22 countries of the Organization for Economic Cooperation and Development [38]. In addition, there is a nationwide shortage of intensivists in Japan [39]. In April 2021, the total number of board-certified intensivists was 2115, whereas the total number of ICU beds was 7132. Only approximately 34% of all ICUs in 2019 met the criteria for resource-rich ICUs with two or more full-time intensivists [32, 40]. Therefore, it is essential to consider that our study is based on results from regions with insufficient critical care capacity. In recent years, many countries have experienced a shortage of ICU beds and growing concern about disparities in access to critical care [13]. A recent study estimated that at least 96 countries and territories, particularly those identified as low- and middle-income, exhibit a density of fewer than 5.0 ICU beds per 100,000 population [13, 14, 16]. Our findings contribute to planning appropriate regional critical care systems for these countries with limited ICU capacities during the pandemic.

Our findings provide insights into the ideal number of critical care capacity per 100,000 individuals at the regional level during the COVID-19 pandemic and the emergence and re-emergence of infectious disease pandemics. A positive linear association existed between resources with ICU beds, resource-rich ICU beds, and intensivists and the incidence of IMV for COVID-19 during the pandemic. In addition, this study revealed that the slope of the increase in the incidence of IMV per additional bed was greater in resource-rich ICU beds than in ICU beds. Notably, the results of this study were from Japan, with insufficient critical care capacity, and it is unclear what would happen if increased critical care capacity was available. Based on our findings for switch point analyses, the ideal number of ICU beds, resource-rich ICU beds, and intensivists per 100,000 individuals during the pandemic is > 7, > 3, and > 2, respectively, to prepare for emerging and re-emerging infectious disease pandemic during normal condition.

In contrast, this study demonstrates that a higher number of HDU beds per population was not associated with a higher incidence of IMV in COVID-19 patients. This may indicate that HDU beds are not a sufficient alternative to ICU beds for COVID-19 patients who require IMV. Mechanically ventilated patients, particularly those with COVID-19, require a high workload from physicians and nursing staff [41, 42]. Evidence indicates inadequate nurse staffing and an increased nurse workload may affect care [43]. Therefore, increasing the number of HDU beds may not be an effective strategy to improve access to care for critically ill COVID-19 patients who require IMV. These findings provide useful information for future healthcare planning for the preparedness for emerging and re-emerging infectious disease pandemics and the allocation of critical care resources.

The ECMO incidence and risk-adjusted mortality results require careful interpretation. Owing to the low incidence of ECMO for COVID-19 patients in Japan, 26/47 (55%) prefectures experienced less than 10 cases of ECMO for COVID-19 during the 3-year study period. Therefore, the association between the incidence of ECMO and critical care capacity has not been adequately evaluated statistically, and further study over extended periods is required. Furthermore, the fact that ECMO was performed at 193 facilities in Japan significantly differs from the five facilities in the United Kingdom, where ECMO cases are consolidated [44]. Our study revealed that the risk-adjusted mortality of COVID-19 patients requiring IMV did not change in patients who were placed on a ventilator, even when critical care capacity increased. Therefore, we could not examine whether increasing critical care capacity improves mortality in newly diagnosed COVID-19 patients who require IMV because those who required critical care but were not ultimately ventilated were excluded from this analysis. Further studies are required to determine whether increased critical care capacity improves mortality in patients newly diagnosed with COVID-19 requiring critical care.

This study has some limitations. First, the observational nature of this study precludes drawing causal inferences from the observed associations. From our study, it remains unknown whether increased critical care capacity improved access to appropriate care for critically ill COVID-19 patients. While a randomized controlled trial would be necessary to establish causal inference, conducting such a trial for the clinical question in this study is unfeasible. Therefore, this study stands as valuable evidence. Second, it is unknown whether hospitals participating in the CRISIS database have registered all IMV cases in their hospitals, which may raise concerns regarding the coverage of ventilator patients in this study. Third, vaccination for COVID-19 significantly reduces progression to a critically ill condition [45], but it is unknown whether the newly diagnosed COVID-19 patients were vaccinated in this study. However, vaccination coverage did not seem to vary significantly among prefectures in Japan [46]; therefore, this can be considered negligible. Fourth, the severity of COVID-19 in newly diagnosed patients and the mortality rate among all COVID-19 patients in each prefecture were unmeasured, potentially introducing bias into this study. Finally, our study focused on Japan, where the population is aging and > 20% of the COVID-19 patients requiring mechanical ventilation are aged > 75 years; therefore, the generalizability of our findings to other countries with different healthcare systems and resource allocations may be limited.

## Conclusions

This study demonstrates that the number of ICU beds, resource-rich ICU beds, and intensivists per 100,000 individuals are positively associated with the incidence of IMV among newly diagnosed COVID-19 patients. Further studies are needed to validate the study’s findings and to investigate the impact of regional critical care capacity on critical care access and outcomes to prepare for emerging and re-emerging infectious disease pandemics.

### Supplementary Information

**Additional file 1: Table S1.** Japanese medical procedure codes used to define the ICU and HDU beds. **Table S2.** Proportion of total ICU and HDU beds in CRISIS-participating hospitals to the total number of ICU and HUD beds in the Survey of Medical Institution in each prefecture. **Table S3.** Correlation matrix for the four indicators of regional critical care capacity. **Table S4.** Switch point analyses for the association between the four indicators of regional critical care capacity and the three study outcomes. **Table S5.** Results of sensitivity analyses with further adjustment for regional-level confounders for the association between the four indicators of regional critical care capacity and the three study outcomes. **Table S6.** Results of sensitivity analyses with multi-level generalized linear models for the association between the four indicators of regional critical care capacity and the risk-adjusted mortality.

## Data Availability

The datasets used and analyzed during the current study are available from the corresponding author upon reasonable request.

## Abbreviations

CI: Confidence interval
COVID-19: Coronavirus disease 2019
CRISIS: CRoss Icu Searchable Information System
ECMO: Extracorporeal membrane oxygenation
HER-SYS: Health Center Real-time Information-sharing System on COVID-19
HDU: High-dependency care unit
ICU: Intensive care unit
IMV: Invasive mechanical ventilation

## References

[1] Nates JL, Nunnally M, Kleinpell R, Blosser S, Goldner J, Birriel B (2016). ICU admission, discharge, and triage guidelines: a framework to enhance clinical operations, development of institutional policies, and further research. Crit Care Med.

[2] Stretch B, Shepherd SJ (2021). Criteria for intensive care unit admission and severity of illness. Surgery.

[3] Ohbe H, Sasabuchi Y, Yamana H, Matsui H, Yasunaga H (2021). Intensive care unit versus high-dependency care unit for mechanically ventilated patients with pneumonia: a nationwide comparative effectiveness study. Lancet Reg Health West Pac.

[4] Ohbe H, Sasabuchi Y, Iwagami M, Ogura T, Ono S, Matsui H (2023). Intensive care unit versus high-dependency care unit for COVID-19 patients with invasive mechanical ventilation. Ann Am Thorac Soc.

[5] Dar M, Swamy L, Gavin D, Theodore A (2021). Mechanical-ventilation supply and options for the COVID-19 pandemic Leveraging all available resources for a limited resource in a crisis. Ann Am Thorac Soc.

[6] Truog RD, Mitchell C, Daley GQ (2020). The toughest triage - allocating ventilators in a pandemic. N Engl J Med.

[7] Griffin KM, Karas MG, Ivascu NS, Lief L (2020). Hospital preparedness for COVID-19: a practical guide from a critical care perspective. Am J Respir Crit Care Med.

[8] Kadri SS, Sun J, Lawandi A, Strich JR, Busch LM, Keller M (2021). Association between caseload surge and COVID-19 survival in 558 U.S. hospitals, march to august 2020. Ann Intern Med.

[9] Critical care statistics. https://www.sccm.org/Communications/Critical-Care-Statistics. Accessed 9 May 2023. In: Society of Critical Care Medicine (SCCM)

[10] Rhodes A, Ferdinande P, Flaatten H, Guidet B, Metnitz PG, Moreno RP (2012). The variability of critical care bed numbers in Europe. Intensive Care Med.

[11] Phua J, Faruq MO, Kulkarni AP, Redjeki IS, Detleuxay K, Mendsaikhan N (2020). Critical care bed capacity in Asian countries and regions. Crit Care Med.

[12] Tu WJ, Liu Y, Zeng X (2023). Critical care capacity during the omicron wave of the COVID-19 pandemic in China: far from enough. Lancet Reg Health West Pac.

[13] Ma X, Vervoort D (2020). Critical care capacity during the COVID-19 pandemic: global availability of intensive care beds. J Crit Care.

[14] Murthy S, Leligdowicz A, Adhikari NKJ (2015). Intensive care unit capacity in low-income countries: a systematic review. PLoS ONE.

[15] Turner HC, Van Hao NV, Yacoub S, Hoang VMT, Clifton DA, Thwaites GE (2019). Achieving affordable critical care in low-income and middle-income countries. BMJ Glob Health.

[16] Salluh JIF, Burghi G, Haniffa R (2021). Intensive care for COVID-19 in low- and middle-income countries: research opportunities and challenges. Intensive Care Med.

[17] Janke AT, Mei H, Rothenberg C, Becher RD, Lin Z, Venkatesh AK (2021). Analysis of hospital resource availability and COVID-19 mortality across the United States. J Hosp Med.

[18] Gibbons PW, Kim J, Cash RE, He S, Lai D, Christian Renne B (2023). Influence of ICU surge and capacity on COVID mortality across U.S. States and regions during the COVID-19 pandemic. J Intensive Care Med.

[19] Bravata DM, Perkins AJ, Myers LJ, Arling G, Zhang Y, Zillich AJ (2021). Association of intensive care unit patient load and demand with mortality rates in US department of veterans affairs hospitals during the COVID-19 pandemic. JAMA Netw Open.

[20] Ogura T, Ohshimo S, Liu K, Iwashita Y, Hashimoto S, Takeda S (2021). Establishment of a disaster management-like system for COVID-19 patients requiring veno-venous extracorporeal membrane oxygenation in Japan. Membranes.

[21] Japan ECM, Onet for COVID-19 (2020). Nationwide system to centralize decisions around ECMO use for severe COVID-19 pneumonia in Japan (Special Correspondence). J Intensive Care Med.

[22] Japan ECM (2020). Mechanical ventilation and extracorporeal membrane oxygenation for acute respiratory failure owing to COVID-19: basic concept. Nihon Shuchu Chiryo Igakukai zasshi.

[23] Japan ECM. Onet for COVID- Survey of Critically Ill COVID-19 Patients in Japan, Managed by the Japan ECMOnet for COVID-19. https://crisis.ecmonet.jp/. Accessed 25 Aug 2023

[24] The Ministry of Health, Labour and Welfare, Japan Visualizing the data: information on COVID-19 infections. https://covid19.mhlw.go.jp/en/. Accessed 25 Aug 2023

[25] The Ministry of Health, Labour and Welfare, Japan survey of medical institutions. https://www.mhlw.go.jp/stf/seisakunitsuite/bunya/0000055891.html. Accessed 9 May 2023

[26] Statistics Bureau, Ministry of Internal Affairs. Communications result of the population estimates. https://www.stat.go.jp/english/data/jinsui/index.html. Accessed 9 May 2023

[27] The Ministry of Health, Labour and Welfare, Japan Health Center. Real-time information-sharing system on COVID-19 (HER-SYS). https://www.mhlw.go.jp/stf/seisakunitsuite/bunya/0000121431_00181.html. Accessed 25 Aug 2023

[28] The Japanese Society of Intensive Care Medicine. icu_hcu_beds.pdf. https://www.jsicm.org/news/upload/icu_hcu_beds.pdf. Accessed 25 Aug 2023. In: Number of ICU beds, HDU beds, and intensivists by prefecture; 2021

[29] Ohbe H, Sasabuchi Y, Kumazawa R, Matsui H, Yasunaga H (2022). Intensive care unit occupancy in Japan, 2015–2018: a nationwide inpatient database study. J Epidemiol.

[30] Prin M, Wunsch H (2014). The role of stepdown beds in hospital care. Am J Respir Crit Care Med.

[31] Prin M, Harrison D, Rowan K, Wunsch H (2015). Epidemiology of admissions to 11 stand-alone high-dependency care units in the UK. Intensive Care Med.

[32] Ohbe H, Sasabuchi Y, Matsui H, Fushimi K, Yasunaga H (2021). Resource-rich intensive care units vs standard intensive care units on patient mortality: a nationwide inpatient database study. JMA.

[33] The Ministry of Health, Labour, and Welfare. Japanese Report from the Subcommittee on Countermeasures to Combat New coronavirus infections, https://www.mhlw.go.jp/stf/shingi/shingi-kousei_127717.html. Accessed 12 Dec 2023

[34] Arin P, Minniti M, Murtinu S, Spagnolo N (2022). Inflection points, kinks, and jumps: a statistical approach to detecting nonlinearities. Organ Res Methods.

[35] Hansen BE (2000). Sample splitting and threshold estimation. Econometrica.

[36] StataCorp. Stata Statistical Software: threshold. https://www.stata.com/manuals/tsthreshold.pdf. Accessed 12 Dec 2023. College Station, TX: StataCorp LP

[37] The Ministry of Health, Labour and Welfare, Japan. Statistics of physicians, dentists and pharmacists 2020. https://www.mhlw.go.jp/toukei/saikin/hw/ishi/20/index.html. Accessed 12 Dec 2023

[38] OECD beyond Containment: health systems responses to COVID-19 in the OECD. https://read.oecd-ilibrary.org/view/?ref=119_119689-ud5comtf84&title=Beyond_Containment:Health_systems_responses_to_COVID-19_in_the_OECD. Accessed 9 May 2023

[39] Shime N (2016). Clinical and investigative critical care medicine in Japan. Intensive Care Med.

[40] Shime N (2022). Resource-rich intensive care units improve health-what is next?. JMA.

[41] Guttormson JL, Calkins K, McAndrew N, Fitzgerald J, Losurdo H, Loonsfoot D (2022). Critical care nurses’ experiences during the COVID-19 pandemic: a US national survey. Am J Crit Care.

[42] Montgomery CM, Humphreys S, McCulloch C, Docherty AB, Sturdy S, Pattison N (2021). Critical care work during COVID-19: a qualitative study of staff experiences in the UK. BMJ Open.

[43] Needleman J, Buerhaus P, Pankratz VS, Leibson CL, Stevens SR, Harris M (2011). Nurse staffing and inpatient hospital mortality. N Engl J Med.

[44] Czapran A, Steel M, Barrett NA (2020). Extra-corporeal membrane oxygenation for severe respiratory failure in the UK. J Intensive Care Soc.

[45] Pilishvili T, Gierke R, Fleming-Dutra KE, Farrar JL, Mohr NM, Talan DA (2021). Effectiveness of mRNA Covid-19 vaccine among U.S. health care personnel. N Engl J Med.

[46] Prime Minister’s Office of Japan COVID-19 vaccines. https://japan.kantei.go.jp/ongoingtopics/vaccine.html. Accessed 25 Aug 2023

