# Supplementary material for: Association between regional critical care capacity and the incidence of invasive mechanical ventilation for coronavirus disease 2019: a population-based cohort study
Source: J Intensive Care. 2024 Jan 30;12:6. doi: 10.1186/s40560-024-00718-2 (PMC10826037; doi:10.1186/s40560-024-00718-2)
Supplement: Supplementary file 1 — Additional file 1: Table S1. Japanese medical procedure codes used to define the ICU and HDU beds. Table S2. Proportion of total ICU and HDU beds in CRISIS-participating hospitals to the total number of ICU and HUD beds in the Survey of Medical Institution in each prefecture. Table S3. Correlation matrix for the four indicators of regional critical care capacity. Table S4. Switch point analyses for the association between the four indicators of regional critical care capacity and the three study outcomes. Table S5. Results of sensitivity analyses with further adjustment for regional-level confounders for the association between the four indicators of regional critical care capacity and the three study outcomes. Table S6. Results of sensitivity analyses with multi-level generalized linear models for the association between the four indicators of regional critical care capacity and the risk-adjusted mortality. [file 40560_2024_718_MOESM1_ESM.docx]

***Supplemental Materials***

**Association between regional critical care capacity and the incidence of invasive mechanical ventilation for coronavirus disease 2019: a population-based cohort study**

Hiroyuki Ohbe, Satoru Hashimoto, Takayuki Ogura, Mitsuaki Nishikimi, Daisuke Kudo, Nobuaki Shime, and Shigeki Kushimoto

**Correspondence to:**

Shigeki Kushimoto

Department of Emergency and Critical Care Medicine, Tohoku University Hospital, 1-1 Seiryo-machi, Aoba-ku, Sendai 980-8574, Japan

E-mail: kussie@emergency-medicine.med.tohoku.ac.jp,

Tel: +81-2-2717-7489, Fax: +81-2-2717-7492

**Supplemental Table 1.** Japanese medical procedure codes used to define the ICU and HDU beds.

|  |  | Japanese |  | Cost |  |  |
| --- | --- | --- | --- | --- | --- | --- |
|  |  | procedure |  | per day, | Intensivist | Nurse-to-patient |
| Type | Subcategory | Code | Description | yen^*^ | staffing | ratio |
| ICU | Resource-rich | A3011 | ICU management fee 1 | 142110 | ≥2 | 1:2 |
| ICU | Resource-rich | A3012 | ICU management fee 2 | 142110 | ≥2 | 1:2 |
| ICU | – | A3013 | ICU management fee 3 | 96970 | – | 1:2 |
| ICU | – | A3014 | ICU management fee 4 | 96970 | – | 1:2 |
| ICU | – | A3002 | Emergency and critical care unit management fee 2 | 106860 | – | 1:2 |
| ICU | – | A3004 | Emergency and critical care unit management fee 4 | 106860 | – | 1:2 |
| ICU | – | A301-4 | Pediatric ICU management fee | 163170 |  | 1:2 |
| HDU | – | A3001 | Emergency and critical care unit management fee 1 | 92500 | – | 1:4 |
| HDU | – | A3003 | Emergency and critical care unit management fee 3 | 92500 | – | 1:4 |
| HDU | – | A301-21 | HDU management fee 1 | 68550 | – | 1:4 |
| HDU | – | A301-22 | HDU management fee 2 | 42240 | – | 1:5 |
| HDU | – | A301-3 | Stroke care unit | 60130 | – | 1:3 |

^*^Cost per day for the first 7 days after admission

ICU, intensive care unit; HDU, high-dependency care unit.

**Supplemental Table 2**. Proportion of total ICU and HDU beds in CRISIS-participating hospitals to the total number of ICU and HUD beds in the Survey of Medical Institution in each prefecture.

|  | Survey of Medical Institution | | |  | CRISIS-participating hospitals | | | | | |
| --- | --- | --- | --- | --- | --- | --- | --- | --- | --- | --- |
|  |  |  | Number of |  |  |  | Number of |  |  | Proportion of |
|  | Number | Number | resource-rich |  | Number | Number | resource-rich | Proportion | Proportion | resource-rich |
|  | of ICU | of HDU | ICU |  | of ICU | of HDU | ICU | of ICU | of HDU | ICU |
|  | beds, | beds, | beds, |  | beds, | beds, | beds, | beds, | beds, | beds, |
| Prefectures | number | number | number |  | number | number | number | % | % | number |
| **Overall** | **7132** | **13546** | **2445** |  | **6186** | **10058** | **2322** | **86.7** | **74.2** | **95.0** |
| Hokkaido | 221 | 647 | 100 |  | 124 | 408 | 70 | 56.1 | 63.1 | 56.1 |
| Aomori | 68 | 71 | 16 |  | 54 | 44 | 16 | 79.4 | 62.0 | 79.4 |
| Iwate | 32 | 116 | 8 |  | 32 | 116 | 8 | 100 | 100 | 100 |
| Miyagi | 126 | 185 | 66 |  | 126 | 169 | 66 | 100 | 91.4 | 100 |
| Akita | 36 | 76 | 16 |  | 36 | 56 | 16 | 100 | 73.7 | 100 |
| Yamagata | 32 | 106 | 6 |  | 22 | 76 | 6 | 68.8 | 71.7 | 68.8 |
| Fukushima | 116 | 171 | 28 |  | 94 | 105 | 18 | 81.0 | 61.4 | 81.0 |
| Ibaraki | 140 | 206 | 16 |  | 118 | 147 | 16 | 84.3 | 71.4 | 84.3 |
| Tochigi | 95 | 192 | 58 |  | 95 | 165 | 58 | 100 | 85.9 | 100 |
| Gumma | 75 | 215 | 53 |  | 75 | 94 | 53 | 100 | 43.7 | 100 |
| Saitama | 327 | 771 | 75 |  | 309 | 551 | 67 | 94.5 | 71.5 | 94.5 |
| Chiba | 400 | 569 | 74 |  | 346 | 467 | 74 | 86.5 | 82.1 | 86.5 |
| Tokyo | 1103 | 1472 | 317 |  | 948 | 1063 | 289 | 85.9 | 72.2 | 85.9 |
| Kanagawa | 480 | 1022 | 84 |  | 451 | 726 | 80 | 94.0 | 71.0 | 94.0 |
| Niigata | 32 | 174 | 8 |  | 24 | 150 | 8 | 75.0 | 86.2 | 75.0 |
| Toyama | 30 | 79 | 8 |  | 20 | 43 | 8 | 66.7 | 54.4 | 66.7 |
| Ishikawa | 52 | 158 | 28 |  | 38 | 73 | 28 | 73.1 | 46.2 | 73.1 |
| Fukui | 37 | 113 | 20 |  | 37 | 87 | 20 | 100 | 77.0 | 100 |
| Yamanashi | 12 | 40 | 12 |  | 12 | 26 | 12 | 100 | 65.0 | 100 |
| Nagano | 105 | 420 | 28 |  | 89 | 300 | 28 | 84.8 | 71.4 | 84.8 |
| Gifu | 74 | 191 | 42 |  | 64 | 171 | 42 | 86.5 | 89.5 | 86.5 |
| Shizuoka | 148 | 378 | 38 |  | 138 | 324 | 34 | 93.2 | 85.7 | 93.2 |
| Aichi | 403 | 768 | 208 |  | 371 | 709 | 198 | 92.1 | 92.3 | 92.1 |
| Mie | 43 | 164 | 6 |  | 37 | 104 | 6 | 86.0 | 63.4 | 86.0 |
| Shiga | 54 | 104 | 12 |  | 42 | 88 | 12 | 77.8 | 84.6 | 77.8 |
| Kyoto | 161 | 355 | 100 |  | 161 | 309 | 100 | 100 | 87.0 | 100 |
| Osaka | 647 | 1077 | 272 |  | 562 | 736 | 270 | 86.9 | 68.3 | 86.9 |
| Hyogo | 371 | 567 | 120 |  | 288 | 432 | 120 | 77.6 | 76.2 | 77.6 |
| Nara | 83 | 160 | 40 |  | 68 | 106 | 40 | 81.9 | 66.3 | 81.9 |
| Wakayama | 61 | 105 | 10 |  | 61 | 93 | 10 | 100 | 88.6 | 100 |
| Tottori | 26 | 82 | 6 |  | 26 | 53 | 6 | 100 | 64.6 | 100 |
| Shimane | 41 | 55 | 28 |  | 37 | 51 | 24 | 90.2 | 92.7 | 90.2 |
| Okayama | 223 | 190 | 77 |  | 178 | 170 | 54 | 79.8 | 89.5 | 79.8 |
| Hiroshima | 101 | 241 | 42 |  | 91 | 197 | 42 | 90.1 | 81.7 | 90.1 |
| Yamaguchi | 84 | 119 | 28 |  | 84 | 71 | 28 | 100 | 59.7 | 100 |
| Tokushima | 34 | 85 | 11 |  | 34 | 85 | 11 | 100 | 100 | 100 |
| Kagawa | 66 | 144 | 28 |  | 66 | 132 | 28 | 100 | 91.7 | 100 |
| Ehime | 78 | 129 | 12 |  | 68 | 79 | 12 | 87.2 | 61.2 | 87.2 |
| Kochi | 54 | 139 | 40 |  | 48 | 130 | 40 | 88.9 | 93.5 | 88.9 |
| Fukuoka | 340 | 748 | 92 |  | 277 | 523 | 92 | 81.5 | 69.9 | 81.5 |
| Saga | 40 | 105 | 18 |  | 40 | 101 | 18 | 100 | 96.2 | 100 |
| Nagasaki | 68 | 142 | 20 |  | 56 | 82 | 20 | 82.4 | 57.7 | 82.4 |
| Kumamoto | 89 | 220 | 51 |  | 79 | 138 | 51 | 88.8 | 62.7 | 88.8 |
| Oita | 44 | 92 | 8 |  | 40 | 63 | 8 | 90.9 | 68.5 | 90.9 |
| Miyazaki | 50 | 57 | 16 |  | 50 | 44 | 16 | 100 | 77.2 | 100 |
| Kagoshima | 90 | 158 | 40 |  | 40 | 53 | 40 | 44.4 | 33.5 | 44.4 |
| Okinawa | 140 | 168 | 59 |  | 130 | 148 | 59 | 92.9 | 88.1 | 92.9 |

ICU, intensive care unit; CRISIS, CRoss Icu Searchable Information System; HDU, high-dependency care unit.

**Supplemental Table 3**. Correlation matrix for the four indicators of regional critical care capacity.

|  |  |  | Resource-rich |  |
| --- | --- | --- | --- | --- |
| Variables | ICU beds | HDU beds | ICU beds | Intensivists |
| ICU beds | 1.000 | – | – | – |
| HDU beds | 0.6485 | 1.000 | – | – |
| Resource-rich ICU beds | 0.6403 | 0.4917 | 1.000 | – |
| Intensivists | 0.4248 | 0.3321 | 0.0578 | 1.000 |

ICU, intensive care unit; HDU, high-dependency care unit.

**Supplemental Table 4**. Switch point analyses for the association between the four indicators of regional critical care capacity and the three study outcomes.

|  | Outcomes | | | | | | | |
| --- | --- | --- | --- | --- | --- | --- | --- | --- |
| Number of regional | Incidence of IMV | |  | Incidence of ECMO | |  | Risk-adjusted | |
| critical care capacity | per 100,000 COVID-19 | |  | per 100,000 COVID-19 | |  | mortality (%) | |
| per 100,000 population | Coef. (95%CI) | P-value |  | Coef. (95%CI) | P-value |  | Coef. (95%CI) | P-value |
| **ICU beds** |  |  |  |  |  |  |  |  |
| Switch point | 6.93 |  |  | 6.13 |  |  | 3.74 |  |
| Region (<switch point) | 2.75 (-0.85, 6.35) | 0.135 |  | 0.04 (-0.66, 0.74) | 0.913 |  | 5.64 (-5.87, 17.2) | 0.337 |
| Region (≥switch point) | -0.83 (-16.4, 14.8) | 0.917 |  | -0.47 (-1.11, 0.17) | 0.153 |  | 0.37 (-1.17, 1.90) | 0.640 |
| **HDU beds** |  |  |  |  |  |  |  |  |
| Switch point | 11.4 |  |  | 14.6 |  |  | 9.94 |  |
| Region (<switch point) | 2.78 (0.20, 5.36) | 0.034 |  | 0.19 (-0.22, 0.60) | 0.371 |  | 0.29 (-2.70, 3.28) | 0.849 |
| Region (≥switch point) | -6.48 (-9.94, -3.02) | 0.000 |  | 0.12 (-0.04, 0.28) | 0.156 |  | 0.50 (-0.98, 1.98) | 0.505 |
| **Resource-rich ICU beds** | |  |  |  |  |  |  |  |
| Switch point | 3.01 |  |  | 2.94 |  |  | 2.94 |  |
| Region (<switch point) | 6.90 (1.52, 12.3) | 0.012 |  | 0.03 (-0.96, 1.01) | 0.960 |  | -1.01 (-3.51, 1.50) | 0.432 |
| Region (≥switch point) | -39.0 (-57.6, -20.4) | 0.000 |  | -2.40 (-3.39, -1.41) | 0.000 |  | 8.86 (7.00, 10.7) | 0.000 |
| **Intensivists** |  |  |  |  |  |  |  |  |
| Switch point | 2.01 |  |  | 1.80 |  |  | 0.87 |  |
| Region (<switch point) | 31.9 (14.8, 49.1) | 0.000 |  | 1.91 (-0.06, 3.87) | 0.057 |  | 40.6 (-14.0, 95.1) | 0.145 |
| Region (≥switch point) | 7.59 (-20.2, 35.3) | 0.592 |  | -2.19 (-4.03, -0.35) | 0.020 |  | 2.68 (-1.95, 7.32) | 0.256 |

ICU, intensive care unit; HDU, high-dependency care unit; IMV, invasive mechanical ventilation; COVID-19, coronavirus disease 2019; ECMO, extracorporeal membrane oxygenation; CI, confidence interval.

**Supplemental Table 5**. Results of sensitivity analyses with further adjustment for regional-level confounders for the association between the four indicators of regional critical care capacity and the three study outcomes.

|  | Outcomes | | | | | | | |
| --- | --- | --- | --- | --- | --- | --- | --- | --- |
| Number of regional | Incidence of IMV | |  | Incidence of ECMO | |  | Risk-adjusted | |
| critical care capacity | per 100,000 COVID-19 | |  | per 100,000 COVID-19 | |  | mortality (%) | |
| per 100,000 population | Coef. (95%CI) | P-value |  | Coef. (95%CI) | P-value |  | Coef. (95%CI) | P-value |
| ICU beds | 3.92 (0.01, 7.83) | 0.050 |  | 0.18 (-0.32, 0.67) | 0.473 |  | -1.15 (-2.93, 0.64) | 0.203 |
| HDU beds | -0.15 (-2.62, 2.32) | 0.904 |  | -0.09 (-0.39, 0.21) | 0.536 |  | -0.65 (-1.73, 0.43) | 0.232 |
| Resource-rich ICU beds | 5.76 (-1.52, 13.1) | 0.118 |  | 0.45 (-0.45, 1.35) | 0.322 |  | 0.57 (-2.77, 3.90) | 0.733 |
| Intensivists | 10.6 (0.16, 21.0) | 0.047 |  | 0.98 (-0.31, 2.28) | 0.132 |  | 1.96 (-2.86, 6.78) | 0.416 |

Outcomes were adjusted with the numbers of emergency physicians, pulmonologists, nurses, and clinical engineers per 100,000 individuals in the regions.

ICU, intensive care unit; HDU, high-dependency care unit; IMV, invasive mechanical ventilation; COVID-19, coronavirus disease 2019; ECMO, extracorporeal membrane oxygenation; CI, confidence interval.

**Supplemental Table 6**. Results of sensitivity analyses with multi-level generalized linear models for the association between the four indicators of regional critical care capacity and the risk-adjusted mortality.

| Number of regional | Risk-adjusted | |
| --- | --- | --- |
| critical care capacity | mortality (%) | |
| per 100,000 population | Coef. (95%CI) | P-value |
| ICU beds | -0.74 (-1.64, 0.16) | 0.109 |
| HDU beds | -0.46 (-1.31, 0.39) | 0.288 |
| Resource-rich ICU beds | -1.67 (-3.81, 0.48) | 0.129 |
| Intensivists | -0.43 (-3.87, 3.00) | 0.806 |

Multi-level generalized linear models of patient-level with mortality as dependent variable, each of four indicators of regional critical care capacity, 5-year age category, sex, body mass index category, and outbreaks of COVID-19 on the day of initiation of IMV as independent variables, prefecture as a random intercept, and identity link function were performed.

ICU, intensive care unit; HDU, high-dependency care unit; CI, confidence interval.
